# Neurofibromatosis Type 1 with Bladder Involvement

**DOI:** 10.1155/2013/145076

**Published:** 2013-07-28

**Authors:** Iyimser Üre, Serhat Gürocak, Ipek Isik Gönül, Sinan Sözen, Nuri Deniz

**Affiliations:** ^1^Department of Urology, School of Medicine, Gazi University, 06500 Ankara, Turkey; ^2^Department of Pathology, School of Medicine, Gazi University, 06500 Ankara, Turkey

## Abstract

Neurofibromatosis type 1 is an autosomal dominant transmitted disease with various clinical manifestations. The bladder is the most commonly affected organ in the genitourinary system. The malignant transformation of the disease is rare, and unlike malign tumors, the treatment option for benign disease is usually conservative. The size and localization of the mass determine the symptoms, most of which are usually not specific. In this paper, we aim to present a 15-year-old patient with neurofibromatosis type 1 with bladder involvement. The clinical presentation and treatment options of this disease are discussed in the light of the literature.

## 1. Introduction

Neurofibromatosis type 1 (NF1) is an autosomal dominant transmitted disease with various clinical manifestations. Alterations of skin pigmentation, iris Lisch nodules, and multiple benign neurofibromas usually constitute the clinical picture. However, patients also have learning disabilities and may develop skeletal abnormalities, vascular disease, central nervous system (CNS) tumors, or malignant peripheral nerve sheath tumors [1]. 

Genitourinary tract is rarely involved in NF1 and less than 80 cases were reported in the literature to date [2]. The bladder is the commonest affected organ in the urinary tract, in which the manifestation of the disease is either as an isolated mass or a diffuse infiltrative process [3]. Approximately less than one-third of these cases are in pediatric population. The most frequent symptoms of these patients are recurrent urinary tract infection, hematuria, and irritative symptoms. Urinary retention and constipation due to the mass compression on intestinal structures are relatively rare findings in NF1 with genitourinary tract involvement [4]. 

We aim to present an adolescent patient with bladder neurofibroma and discuss our case with the literature.

## 2. Case History

**Figure 1:**
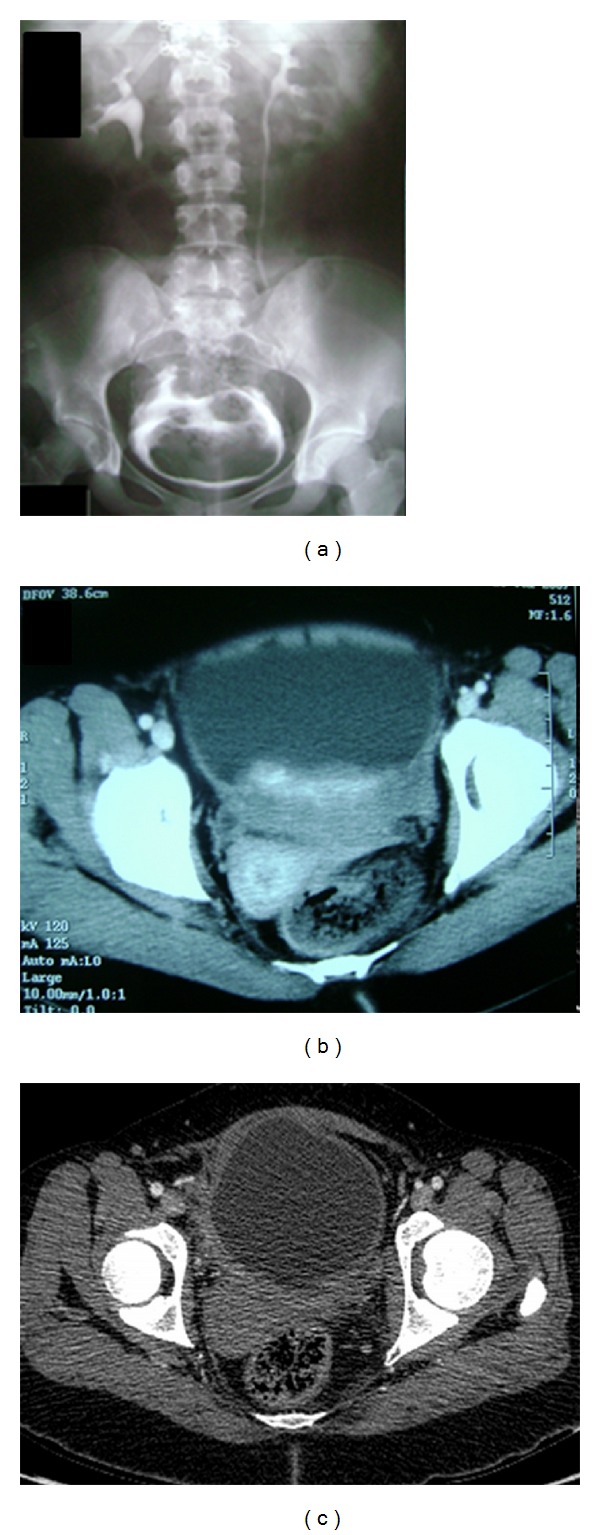
Intravenous pyelography of the patient before operation. A large filling defect can be seen in the bladder (a). A CT image showing a heterogeneous, contrast enhancing mass at the posterior wall of the bladder extending through the ureteral orifices (b). Five years after operation. CT image shows no radiological evidence of the disease (c).

A 15-year-old girl referred to our clinic with the symptoms of hematuria, dysuria, and urinary frequency for the last 6 months. Her medical history was unremarkable. In physical examination, there were axillary freckling and multiple “café au lait” spots around her body with more than two subcutaneous nodules. No neurologic deficiency was observed in her examination. A large filling defect was demonstrated in the urinary bladder without any abnormality in the upper urinary system with IVP (Figure 1(a)). Computerized tomography (CT) showed a 9 cm heterogeneous contrast enhancing mass in the posterior wall of the bladder surrounding two ureteral orifices (Figure 1(b)).

Tissue sample was obtained with transurethral resection of indentation of a 5 × 5 cm mass on the trigon of the bladder. The pathologic examination of the sample was reported as “ganglioneuroma.” Cranial magnetic resonance imaging revealed multiple hamartomatous lesions without any ophtalmic pathology. According to the clinical findings, the patient was diagnosed with neurofibromatosis type 1. 

**Figure 2:**
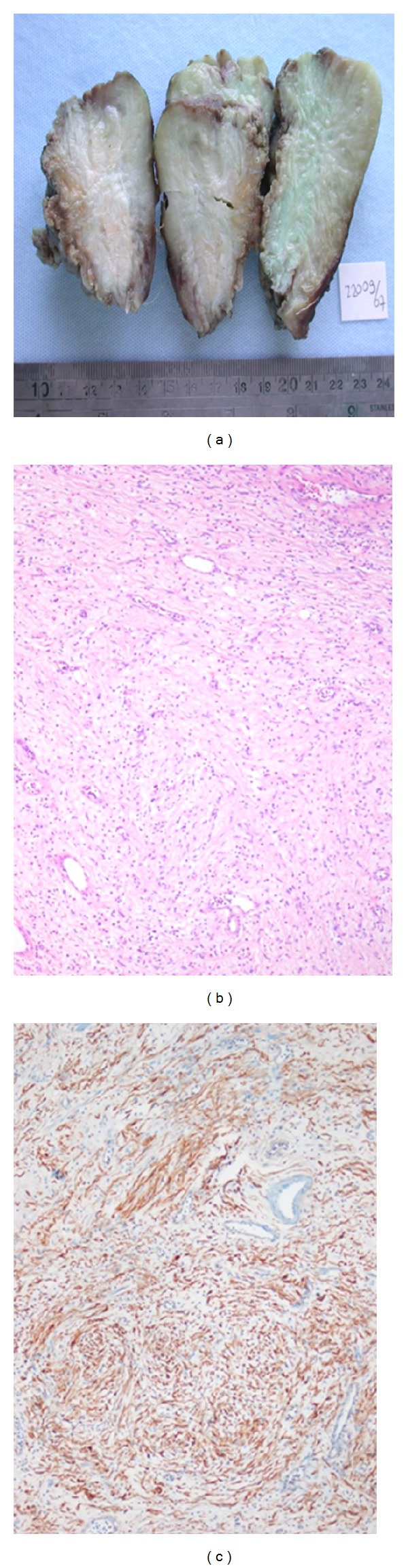
Gross specimen of partial cystectomy showing a diffuse infiltrative mass in the wall of the bladder (a). Light microscopy showed a moderately cellular lesion composed of spindle cells with poorly defined, palely eosinophilic cytoplasm and tapering nuclei, admixed with mononuclear inflammatory cells H&E ×100 (b). Immunostaining showing diffuse and strong positivity of the lesion with S-100 protein, streptavidine biotin peroxidase ×100 (c).

The patient underwent laparotomy, and subtotal resection of the bladder wall together with bilateral ureteral re-implantation was performed. The pathologic examination of the diffusely infiltrative bladder mass showed a moderately cellular lesion composed of S-100 (+) spindle cells with poorly defined, palely eosinophilic cytoplasm and tapering nuclei, admixed with mononuclear inflammatory cells, and it was reported as “neurofibroma” (Figure 2). 

The patient is being followedup for 5 years, and the control computed tomography shows no evidence of the disease (Figure 1(c)).

## 3. Discussion 

Neurofibromatosis 1 (NF1) is an autosomal dominant disease caused by heterozygous mutations of the NF1 gene. NF1 is characterized by wide variability of clinical forms, and most patients with NF1 have only milder manifestations of the disease, such as pigmentary lesions and Lisch nodules [1]. 

Neurofibromas can involve genitourinary tract such as penis, clitoris, prostate, urethra, testis, spermatic cord, and ureter. However, NF most commonly affects the bladder [5]. The condition is more common in males than females by a ratio of 3 : 1. Ross was the first to report a case of a neurofibroma of the bladder with malignant transformation in a neurofibromatosis type 1 patient [6]. Malignant transformation in generalized neurofibromatosis has been reported in 12–29% of patients [7]. The most common symptoms of the disease are hematuria, dysuria, and mainly irritative complaints. However, in some cases, dysfunctional voiding or even urinary retention related to mass localization may occur. Krishna et al. reported a patient with neurofibromatosis suffering urinary retention as an initial symptom [8]. Our patient only had hematuria and complaints of irritative symptoms at the beginning, which can easily be disregarded clinically. 

A systemic physical examination may facilitate the diagnosis of NF if café-au lait spots and superficial neurofibromas are present and recognized. Imaging techniques are useful for assessing the manifestations of neurofibromatosis especially for the lesions in abdominopelvic and cranial regions. Apart from the hamartomatous lesions in the brain, no systemic lesion was found in our patient's scans. These techniques are useful in either early detection of neurofibromas or a possible malignant transformation, suspicious radiological signs of malignancy include an asymmetry in size and heterogeneity in CT attenuation [9]. 

Our patient had a large asymmetric and heterogeneous mass in CT and MRI scans of the abdomen; however, no malignant transformation was detected in the pathologic examination. In radiological investigations, bladder involvement can only be seen as a thickening of the bladder wall or a large mass just like our patient. 

Regarding the variability of clinical signs, symptoms, and organ involvement in this disease, every patient must be evaluated individually in terms of treatment modalities. Those presenting with irritative voiding symptoms should be treated with anticholinergic medications for as long as possible. If the disease progresses during this conservative treatment, most patients develop obstructive voiding symptoms due to the mass effect of the lesion and may require surgery. Even urinary diversion can be considered as a surgical option for these patients. In the case of a ureteral obstruction and hydronephrosis, ureteral reimplantation or partial cystectomy can be a choice of surgery [10]. Frozen section histologic examination of the distal ureter is required to avoid a residual involved tissue. When diversion is necessary, surgical resection of the bladder and the tumor should be considered. Removal of the mass allows for more accurate pathological diagnosis; thus, a possible cure of the disease can be achieved. In our patient, we performed a subtotal resection of the bladder and reimplanted the ureters due the resection of the trigone. 

As a conclusion, the prognosis of neurofibromas is generally very good with a very rare malignant transformation rate. Most cases reported in the literature have been treated with local excisions. If asymptomatic disease is present, surveillance may be the treatment choice. In our patient, despite having a very large tumor, subtotal excision of the bladder with negative surgical margins provided a 5-year progression-free survival.
